# Screening the genome to detect an association with hypertension

**DOI:** 10.1186/1471-2156-4-S1-S63

**Published:** 2003-12-31

**Authors:** Elizabeth J Atkinson, Mariza de Andrade

**Affiliations:** 1 Division of Biostatistics, Department of Health Sciences Research, Mayo Clinic, Rochester, Minnesota, 55905, USA

## Abstract

We report tree-based association analysis as applied to the two Framingham cohorts and to the first replication of the simulated data obtained from the Genetic Analysis Workshop 13. For this analysis, familial association is ignored. The two endpoints examined are hypertension status at initial visit and time-to-hypertension, using a censored data approach. Although linkage association has previously been reported with hypertension, we found no association using the tree-based methodology.

## Background

The Framingham Heart Study is a rich data set filled with information about hypertension status in a population-based cohort observed over an extended period of time. As shown by Levy et al. [1] and Hunt et al. [2], there is strong indication that there is a genetic component to this disease, thus making the data ideal for exploring the strength of tree-based models in detecting similar results. Tree-structure models can be accurate classifiers (binary outcome) and predictors (quantitative outcome), and often yield better understanding to the underlying structure of the data relationship [3]. The use of tree-structure models is advantageous because no assumptions are necessary to explore the data structure and to derive parsimonious models. These models handle data of complex structure and missing data at each node; interactions are part of the tree building process. By using tree-structure methods, we set out to identify homogeneous groups by partitioning the genetic and environmental data using the recursive partitioning algorithm. As shown in Zhang and Bonney [4], classification trees can be used to correctly identify disease alleles. The purpose of this paper is to determine how well the recursive partitioning methodology detected a genetic association using two different measures of hypertension status: hypertension status at the initial visit and a censored data approach, where time is measured on the age scale.

## Materials and Methods

### Data

Our analysis focused on the two Framingham cohorts and the first replicate of the simulated cohorts. Cohort 2 was also used for validation of results obtained from the Cohort 1 analyses. Answers were obtained for the simulated data. In the Framingham study partial data were available on 1213 of the original (largely unrelated) Cohort 1 subjects and 1668 of the original (familial) Cohort 2 subjects. However, in Cohort 1 only 32% of the subjects had genetic marker data; in Cohort 2 the number was much higher (78%) though there were still a large number of uninformative subjects. Although the tree models can handle missing data through the use of surrogate variables, we felt that the large number of observations with completely missing genetic data warranted their deletion. In addition, the age ranges of the two cohorts varied, hence we focused the analysis on the 390 Cohort 1 subjects and the 726 Cohort 2 subjects with genetic marker information who were between 30 and 55 years of age at the initial visit. Similar limiting of the simulated data resulted in 346 Cohort 1 subjects and 1060 Cohort 2 subjects. We defined two primary response variables based on the available phenotype data and models were fit separately for each of the two cohorts. We first looked at hypertension status at the baseline visit (22% of Framingham Cohort 1, 20% of Framingham Cohort 2, 6% of Simulated Cohort 1, and 8% of Simulated Cohort 2). Three Framingham Cohort 2 subjects who were being treated for hypertension at the baseline visit but were not diagnosed with hypertension were removed from the analysis. Our second end-point used a censored "time-to-hypertension" approach, in which age was used as the time scale. Subjects entered the risk set at their first visit and exited the risk set when they were lost to follow up (censored) or were diagnosed with hypertension. For those subjects that did not already have hypertension at their first visit, 52% of Framingham Cohort 1 and 29% of the Framingham Cohort 2 went on to develop the disease by age 55. The simulated cohorts each had estimates around 30% at age 55. Subjects who started treatment for hypertension before diagnosis of hypertension were censored at the time of treatment (this occurred in less that 2% of the subjects in each cohort). The 398 genetic markers were used as predictors along with the environmental variables of age, sex, cigarettes per day, body mass index, and alcohol consumption. The markers were expanded so that every allele of every marker created a new allelic variable. For instance, chromosome 1, marker 1, allele 1 (c1m1a1) could have the values 0, 1, or 2 depending on how many copies of the mutation an individual carried. This resulted in the creation of over 4400 predictors.

### Statistical methods

Construction of trees using recursive partitioning necessitates defining a splitting rule and pruning rule. Splitting rules are used to examine all possible splits of the full group of subjects (root node) and to identify the variable at each level that produces the most homogenous children. For classification endpoints like "hypertension at visit one", impurity is used to determine the best split. Impurity, or the diversity, of a node A can be written as , where C is the number of end-point classes (in our case 2), p_*iA *_is the proportion of those in node A that belong to class *i*, and *f *is some impurity function. If node A is pure then I(A) = 0. For the classification end-point "hypertension at visit one" we used the impurity function called the Gini index where *f*(p) = p(1 - p) and we used the split which maximized the impurity reduction Δ*I *= *p*(*A*_*Parent*_)*I*(*A*_*Parent*_) - *p*(*A*_*Left*_)*I*(*A*_*Left*_) - *p*(*A*_*Right*_)*I*(*A*_*Right*_). For the censored "time-to-hypertension" end-point we used a splitting criterion which is equivalent to a likelihood ratio test for two Poisson groups: *D*_*Parent *_- (*D*_*Left *_+ *D*_*Right*_). The deviance , where c_*i *_is the observed event count for observation *i*, t_*i *_is the scaled observation time, and  is the predicted rate of the node. The time variable is modified slightly using exponential scaling to get a straight line curve for log(survival) under a parametric exponential model [5]. This is equivalent to the local full likelihood tree model by LeBlanc and Crowley [6].

For pruning we chose to use a fixed complexity parameter of 5% and the 1-SE rule. The complexity parameter α is a measure of improvement in the tree impurity. Heuristically, the tree building process can be compared to forward step-wise regression, where variables (splits) are made until the F-test of the remaining variables fails to achieve some level of α. An α level of 5% suggests that further splits will add less than 5% to the overall fit of the tree. The 1-SE rule uses cross-validation, which involves randomly partitioning the original samples into 10 fitted sub-samples and computing an average misclassification rate for each sub-tree (corresponding to the different splits). The sub-tree with the smallest average misclassification rate is identified, as are all sub-trees that have an average misclassification rate within one standard error of this smallest rate (also obtained from the cross-validation). The simplest model is picked. Thus, we identified a sub-tree that provides the least complexity and the least misclassification of subjects. All recursive partitioning models were fit using the SPLUS library rpart [5], a package that closely resembles the original CART package [3].

## Results

### Hypertension status at Visit 1

Analysis of the Framingham Cohort 1 data examining "hypertension at visit one" as built to the complexity parameter of 5% includes the environmental variables age and body mass index (BMI), and 11 different markers: c10m5a5, c12m17a7, c17m7a7, c19m8a1, c1m32a4, c2m21a3, c2m21a6, c2m2a11, c5m20a1, c6m12a7 and c6m13a7. No variables remain in the model after pruning using the 1-SE rule. Results are similar when only marker data is used or when only a subset of the marker data is used (using alleles that appear > 1% or < 99% of the time results in 30% fewer allele variables). Analysis of the Framingham Cohort 2 data resulted in similar conclusions, with no variables remaining after pruning with the 1-SE rule.

Table 1 summarizes the results when the complexity parameter is set to 5%. Although the classification error is marginally improved with the splits (using no splits, the misclassification is 22.1% and it is 17.4% with five terminal-nodes when looking at a model with only marker data), the mean cross-validation error is higher (in this case 26.9%). The misclassification rate, when the model from Cohort 1 is applied to Cohort 2, also worsened (20.2% to 22.7% in the marker only case). Results were similar for the simulated data (see Table 2), including the analysis that only included markers that were close to the underlying genes that were used in the creation of the simulated data. Careful inspection of the individual splits illustrates further the instability of these models. In most cases the improvement gained by using a given allele variable is only marginally better than the improvement gained by a totally different allele (often from a different marker or chromosome). Sometimes the "improvement" only differed in the 5^th ^decimal point.

### Time-to-hypertension

**Figure 1:**
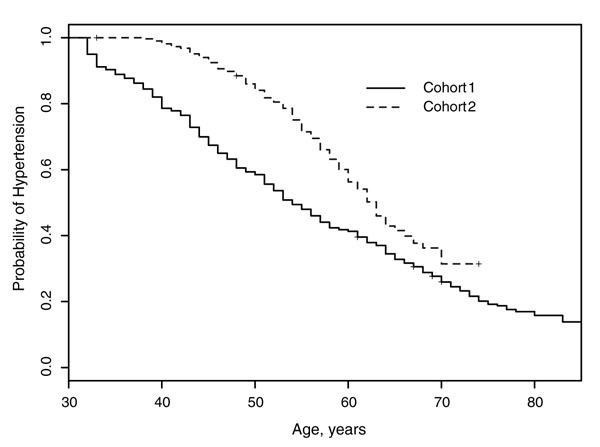
**Time to hypertension in the Framingham cohorts **Survival curves for Cohorts 1 and 2 of the Framingham study, using a censored "time-to-hypertension" approach, where age is used as the time scale (*p *= 0.001).

As is illustrated in Figure 1, the probability of developing hypertension clearly increases as age increases. Thus, an end-point accounting for this change over age should result in a better endpoint. However, none of the "time-to-hypertension" Framingham models improved the node purity by at least 5%. Examination of the cross-validation errors when the trees were grown to their maximum size (complexity parameter set to zero) indicated that no splits were necessary. Results were similar for the simulated data.

## Discussion

Although the recursive partitioning approach should be ideal for detecting sub-populations of individuals that have hypertension, we were unable to detect any association between the hypertension status and the 398 genetic markers. Similarly, because hypertension is highly age-related the end-point using a censored "time-to-hypertension" approach, where age was used as the time scale, should have been useful for separating out subjects who perhaps had earlier onset of the disease. Neither methodology found any consistent positive results. A large proportion of the subjects did not have genetic marker data and were thus deleted (68% of the Framingham Cohort 1 subjects and 22% of the Cohort 2 subjects) whereas in a familial analysis some of the markers could have been estimated from family members. In the Framingham Cohort 1, those without marker data were on average older, heavier, smoked more, drank more and had, at baseline, more hypertension (41.5% vs. 22.1%). The same patterns were also seen in Cohort 2, though the differences were not as great. Because the missing patterns are significantly related to hypertension status, this probably introduced bias into the study, perhaps making meaningful associations more difficult to find. Another problem could be that hypertension is too common a condition for study using this methodology (the cumulative incidence at age 80 is over 80% (Figure 1). The censoring of data when hypertension treatment preceded diagnosis occurred in less than 10% of the cases, but might have marginally influenced the results. However, it is unclear how those subjects should be coded since time to diagnosis would surely be influenced by treatment. Finally, perhaps sample size was an issue, especially in Cohort 1, in which 390 subjects were used for the case/control status and 304 subjects were used for the "time-to-hypertension" endpoint.

## Figures and Tables

**Table 1:** Misclassification rates of the Framingham Cohort 1 models predicting hypertension at visit 1.

**Model^A^**	**Number of End-nodes**	**Cohort 1 Observed Error**	**Cohort 1 Cross-validation**	**Cohort 1 Model Applied to Cohort 2**
1) No Splits	1	22.1%	22.1%	20.2%
2) All M & E	3	18.5%	23.1%	21.6%
3) All M	5	17.4%	26.9%	22.7%
4) All E, subset of M	3	18.5%	23.1%	21.6%

^A^1) no splits; 2) all allelic markers and environmental factors; 3) all allelic markers; and 4) all environmental factors but only those allelic markers that occur at least 1% of the time or less than 99% of the time. All models shown have a complexity parameter of 5%.

**Table 2:** Misclassification rates of the simulated (Replication 1) Cohort 1 models predicting hypertension at visit 1.

**Model^A^**	**Number of End-nodes**	**Cohort 1 Observed Error**	**Cohort 1 Cross-validation**	**Cohort 1 Model applied to Cohort 2**
1) No Splits	1	6.4%	6.4%	7.9%
2) All M & E	3	5.5%	6.6%	10.1%
3) All M	3	5.5%	6.6%	10.1%
4) All E, subset of M	3	5.5%	6.6%	10.1%
5) All E, handpicked M	1	6.4%	6.4%	7.9%

^A^1) no splits; 2) all allelic markers and environmental factors; 3) all allelic markers; 4) all environmental factors but only those allelic markers that occur at least 1% of the time or less than 99% of the time; and 5) all environmental factors but only those allelic markers that occur near the region of the underlying disease genes. All models shown have a complexity parameter of 5%.
